# Resilience in Incident Hemodialysis: Characterization and Outcome Prediction

**DOI:** 10.1093/geroni/igaa057.2713

**Published:** 2020-12-16

**Authors:** Karen Bandeen-Roche, Jiafeng Zhu, Deidra Crews, Mara McAdams-DeMarco, Brian Buta, Ravi Varadhan, Jeremy Walston

**Affiliations:** 1 Johns Hopkins Bloomberg School of Public Health, Baltimore, Maryland, United States; 2 School of Public Health, Johns Hopkins University, Baltimore, Maryland, United States; 3 Johns Hopkins University, Baltimore, Maryland, United States

## Abstract

The Resiliency in Dialysis Initiation (ReDI) Study aims to develop physical resilience signatures in older adults initiating hemodialysis. Study design—comprising a pilot, confirmatory study, and secondary data analyses—will be presented. So also will a method for characterizing resilience phenotypes—using mixed-model analysis of SF-36 subscale trajectories—among participants of age 55 and older who had undergone hemodialysis in the Choices for Healthy Outcomes in Caring for ESRD study (n=485). Analyses revealed stable, improving, and declining phenotypes. In Cox models, both baseline phenotypic status and trajectory type predicted mortality after adjusting for age, CVD status, and CHF (global Wald test for trajectory type P-value=0.020 for vitality; 0.030 for general health). These analyses evidence usefulness of resilience phenotypes as markers of adverse outcome risk and foreshadow application to novel ReDI data.

